# The Management of Infancy, Physiological and Moral

**Published:** 1871-11

**Authors:** 


*The Management of Infancy Physiological and Moral*; intended chiefly for the use of parents. By Andrew Combe, M.D. New York: D. Appleton & Co, 1871.

This is a popular well written and forcible presentation of the claims of young humanity upon the more enlightened portion of mankind. It shows how defective has been the moral and physical care of infancy, and by what steps it has been, and is being, raised to a more enlightened and humane standard. The work claims the attention of all true lovers of humanity, either in its early stages or more advanced periods.

